# Physical fitness and dementia risk in the very old: a study of the Lothian Birth Cohort 1921

**DOI:** 10.1186/s12888-018-1851-3

**Published:** 2018-09-04

**Authors:** Ruth A. Sibbett, Tom C. Russ, Mike Allerhand, Ian J. Deary, John M. Starr

**Affiliations:** 1 Alzheimer Scotland Dementia Research Centre, The University of Edinburgh, 7 George Square, Edinburgh, EH8 9JZ UK; 2 Centre for Cognitive Ageing and Cognitive Epidemiology, The University of Edinburgh, Edinburgh, UK; 3 Department of Psychology, The University of Edinburgh, Edinburgh, UK; 4 Centre for Dementia Prevention, The University of Edinburgh, Edinburgh, UK

**Keywords:** Terms, Dementia, Cohort studies, Risk factors, Fitness

## Abstract

**Background:**

Previous studies have demonstrated that individual measures of fitness – such as reduced pulmonary function, slow walking speed and weak handgrip – are associated with an increased risk of dementia. Only a minority of participants included in these studies were aged over 80. The aim of this study was therefore to investigate the association between physical fitness and dementia in the oldest old.

**Methods:**

Subjects (*n* = 488) were enrolled in the Lothian Birth Cohort 1921 and aged 79 at baseline. Dementia cases arising after enrolment were determined using data from death certificates, electronic patient records and clinical reviews. Fitness measures included grip strength, forced expiratory volume in 1 s (FEV_1_) and walking speed over 6 m, measured at 79 years. Dementia risk associated with each fitness variable was initially determined by logistic regression analysis, followed by Cox regression analysis, where death was considered as a competing risk. *APOE* ε4 status, age, sex, height, childhood IQ, smoking, history of cardiovascular or cerebrovascular disease, hypertension and diabetes were included as additional variables. Cumulative incidence graphs were calculated using Aalen-Johansen Estimator.

**Results:**

Although initial results indicated that greater FEV_1_ was associated with an increased risk of dementia (OR (odds ratio per unit increase) 1.93, *p* = 0.03, *n* = 416), taking into account the competing risk of mortality, none of the fitness measures were found to be associated with dementia; FEV_1_ (HR (hazard ratio per unit increase) 1.30, *p* = 0.37, *n* = 416), grip strength (HR 0.98, *p* = 0.35, *n* = 416), walking speed (HR 0.99, *p* = 0.90, *n* = 416). The presence of an *APOE* ɛ4 allele was however an important predictor for dementia (HR 2.85, *p* < 0.001, *n* = 416). Cumulative incidence graphs supported these findings, with an increased risk of dementia for *APOE* ɛ4 carriers compared with non-carriers. While increased FEV_1_ was associated with reduced risk of death, there was no reduction in risk for dementia.

**Conclusions:**

In contrast to previous studies, this study found that lower fitness beyond age 79 was not a risk factor for subsequent dementia. This finding is not explained by those with poorer physical fitness, who would have been more likely to develop dementia, having died before onset of dementia symptoms.

**Electronic supplementary material:**

The online version of this article (10.1186/s12888-018-1851-3) contains supplementary material, which is available to authorized users.

## Background

Overall physical fitness may be considered as the capacity of an individual’s body to undertake varying degrees of physical activity. As such, physical fitness can reflect a person’s ability to undertake the physical activities required to achieve day-to-day function. Overall physical fitness is comprised of a number of factors; the most important of which are probably muscular strength, muscular endurance, and cardiorespiratory or cardiovascular endurance. In the investigation of associations between fitness and disease, test measures should therefore reflect at least one of these components. Within the literature, tests of lung function, walking speed and grip strength are frequently used to measure physical fitness. Such measures reflect the components of physical fitness described above, with lung function tests providing a measure of cardiorespiratory function, grip strength providing a measure of muscular strength and walking speed providing a measure of both muscular endurance and cardiorespiratory endurance. A latent trait representing physical fitness can be extracted from these variables which is significantly associated with non-pathological cognitive change in this cohort of older adults at age ~ 80 years [1].

By examining the relationship between such physical fitness measures and disease, studies can determine whether fitness levels may be considered as a risk factor for disease. With widely recognised risk factors for dementia – such as age and *APOE* ε4 allele status – being fixed and unchangeable, identifying potentially modifiable risk factors would be of considerable value in contributing to the development of strategies aimed at reducing disease incidence. Such strategies are of particular importance in dementia, given the lack of cure or universally effective treatments. Several previous studies have therefore explored the potential association between physical fitness and dementia. Such studies have demonstrated that reduced pulmonary function [2–8], slow walking speed [9–13] and weak handgrip [9, 10, 14, 15] are associated with an increased risk of dementia. One might hypothesise that these findings reflect the importance of maintaining an optimal blood supply to the brain [16]. Specifically, if an individual has poor lung function, it is possible that blood oxygenation levels are below those required to maintain brain health. Furthermore, slow walking speed may be due to cardiovascular deficiencies and if the heart and circulatory system are struggling to meet the requirements of other areas of the body, it is possible that the brain is experiencing similar deficiencies. While none of these deficiencies may be dramatic enough to result in immediate clinical concern, it is possible that slight inadequacies that are present over a period of time could result in changes to brain structure or function. It is also possible that with increased fitness comes a reduction in other potential risk factors for dementia, such as hypertension, diabetes and increased body mass index [16]. The documented association between physical fitness and normal cognitive ageing [1], together with evidence that physical activity – which correlates with physical fitness – is a risk factor for dementia in generally younger populations [17], suggests that there is good a priori rationale to investigate whether such risk persists into the ninth decade and beyond.

Only a minority of participants included in the aforementioned studies were aged over 80 years. Those studies with an older mean participant age may involve participants from a wide age range (for example: mean age 80.3 years, age range 54–100 years) [14] and it is not therefore possible to draw any inferences regarding the relationship between fitness and dementia in oldest age given the potential influence of those younger participants included in the analyses. Based on current evidence, it remains unclear whether the patterns of association identified in early old age persist into oldest age. Within the literature, studies looking specifically at the oldest-old are typically fewer in number, likely as the result of difficulties in recruitment and retention of study participants, for reasons including co-morbidity and mortality. Understanding how the risk factor profile for dementia changes with age is vital in designing successful preventative strategies. In 2017, the number of persons aged 79 years or older in the UK was estimated to be 3,635,993; 5.5% of the total population [18]. As life expectancy increases and the global population ages, the number of persons reaching oldest-age are expected to grow, reinforcing the importance of understanding patterns of disease in this growing section of the population through further research.

This study considers the participants of the Lothian Birth Cohort 1921 (LBC1921), who were recruited at age 79 years and have undergone follow-up for dementia into their 90s. While a previous study of the LBC1921 had identified an association between superior physical fitness at age 79 years and improved cognitive aging [1], the effect of fitness at age 79 years on the development of dementia had not been investigated in this cohort. Measures of fitness collected at baseline (grip strength, time to walk 6 m and forced expiratory volume in 1 s (FEV_1_)) reflected those commonly used within the literature and would therefore allow for comparisons with previous findings. As such, the study of physical fitness measures and dementia within this detailed, narrow-age cohort would be well-suited to add evidence, specific to the oldest-old, to the literature. Given the frequency of death within a cohort of advanced age we recognised the possibility that this may influence our findings. Such consideration was particularly important given that associations between reduced grip strength, reduced pulmonary function, reduced walking speed and increased risk of death have been well documented [19, 20]. We therefore planned to consider death as a competing risk in our analyses.

The primary aim of this study was therefore to explore the association between three measures of physical fitness – grip strength, lung function and walking speed – and subsequent dementia in persons aged over 79 years.

## Methods

### Participants

Participants were members of the Lothian Birth Cohort 1921 (LBC1921), which is described more fully elsewhere [21]. Most had taken part in general intelligence testing at age 11, as part of the Scottish Mental Survey 1932 (SMS1932) [22]. Childhood cognitive test scores were available for 89.6% of those LBC1921 participants who attended baseline testing. Missing childhood cognitive data was likely to have resulted from school absence on the day of testing (due to sickness, for example), or be because the test results for some schools in Fife (a region neighbouring Lothian) were not found. From 1999, SMS1932 participants were traced and recruited for follow-up in later life, with the aim of investigating normal cognitive ageing [23]. Five-hundred and fifty participants, mostly from the Lothian area of Scotland, enrolled in the study and attended the first wave of testing. Participants were aged approximately 79 years at baseline. Surviving participants who continued to take part in the study were re-tested at four subsequent test waves; at ~ 83, 87, 90 and 92 years of age (approximately 3-yearly intervals). This interval was planned as the period over which the authors expected to be able to detect significant changes in some cognitive test scores, while balancing this against attrition. At each wave, data were collected by questionnaire and in-person testing. Participant deaths were ascertained prospectively, at regular intervals, with details supplied by the General Registrar’s Office, Scotland. The Lothian Research Ethics Committee (test waves 1–3) and the Scotland A Research Ethics Committee (test waves 4–5) provided ethical approval for the study. From wave 4, attending participants provided consent for data linkage and access to health records.

### Fitness variables

The three fitness measures included in this study were 6-m walk time, grip strength and forced expiratory volume in 1 s (FEV_1_) [1]. Walking speed, grip strength and FEV_1_ have all been shown to be valuable in evaluating physical and functional capacity [24–27]. Six-metre walk time was taken as the time in seconds for a participant to walk a measured length of 6 m, at a normal walking pace. Subjects were permitted to utilise any habitually used walking aid, while those who were unable to walk six metres would not participate in the test. A Jamar Hydraulic Hand Dynamometer was used to measure grip strength in kilograms, in the dominant hand; the best of three trials was recorded. FEV_1_ was measured using a microspirometer, in units of litres per second; the best of three attempts was recorded. Taking three measurements is in line with guidance for clinical practice [28].

### Additional variables

The analyses would also include variables deemed to be of potential importance with regard to their association with either physical fitness or dementia. In a previous study of the risk factors for dementia in LBC1921, positive *APOE* ɛ4 carrier status was found to increase risk, whereas a history of hypertension was found to reduce risk [29]. These variables were therefore included, along with the following important control variables: age, sex, height, age 11 IQ, smoking status, history of cardiovascular or cerebrovascular disease and history of diabetes. Age, sex and height are all key control variables given the direct impact on physical strength and fitness. Similarly, a history of cardiovascular or cerebrovascular disease and diabetes history were included given the potential association with physical fitness and dementia. When such conditions impact a person’s ability to partake in exercise, fitness is likely to be adversely affected. The inclusion of age 11 IQ as a control variable is important given the known association with dementia [30] and other fitness-related factors [1, 31]. We also considered the potential impact of smoking status given the likely association with physical fitness, and lung function in particular. Whilst other factors were potentially associated with fitness, we could not include all possible risk factors, as this would have led to multiple hypotheses testing. We therefore adopted the hypothesis-driven approach described above, based on previous findings.

### Data collection procedures

*APOE* ɛ4 status was determined using genomic DNA isolated from participants’ venous blood. Any history of hypertension, diabetes, cerebrovascular or cardiovascular disease, smoking status (ex-, current-, or never-) and sex were self-reported by participants at the first wave of testing. A positive history of cerebrovascular or cardiovascular disease included those reporting previous stroke, transient ischaemic attack, myocardial infarction, angina, coronary artery bypass graft and angioplasty procedures and those who reported a positive history but did not specify the specific nature. A positive history of diabetes included those reporting any history of diabetes (any type or type unspecified). Age at baseline was taken from the number of days between date of birth and date of attendance at wave 1 testing. Age 11 IQ was calculated based on the results of the Moray House Test (MHT) no.12, the general intelligence test taken by participants at age 11 as part of SMS1932 [21]. MHT scores were corrected for age at testing, then converted to IQ-type scores with a standardised mean score of 100 (SD 15). Height in centimetres was measured using a SECA stadiometer.

### Dementia ascertainment

Follow-up for the purpose of dementia ascertainment has been described previously and involved the retrospective collection of evidence, from enrolment to age 95 years [29]. Data were collected from death certificates, electronic hospital records, and clinical reviews. Each death record available by 30th June 2016 was examined for evidence of dementia or cognitive impairment. Electronic hospital records and psychiatric records were accessed for participants who were willing and able to consent to data linkage. Any evidence for dementia or cognitive impairment since enrolment in the study – whether a confirmed diagnosis or evidence for a diagnosis – was recorded. Data were collected from electronic medical records to the 16th May 2016. Additional information was available for 26 participants who underwent clinical review by the authors (TCR, JMS), either in the NHS or research setting. Information from such reviews was collected up to 15th December 2016, when all of the evidence from each source was considered at a final dementia diagnosis consensus meeting (RAS, TCR, JMS). The evidence was examined against a previously described list of criteria for ‘probable’ or ‘possible’ dementia diagnosis [29]. Using these criteria, the meeting agreed upon the presence of a diagnosis and the subtype. Any disagreement was resolved through discussion.

### Event variables

The events considered in these analyses would be dementia and death. Determination of these outcomes was as described above. Time to death was taken as the number of days between the date of attendance at wave 1 testing and date of death. For those who did not die, the censoring time was taken as the number of days between wave 1 testing and a date beyond that last date of data collection for any participant. Time to dementia was taken as the number of days between the date of attendance at wave 1 testing and the first date that a dementia diagnosis was noted in any of the available sources. If dementia was recorded on a death certificate only, and no duration was noted, the diagnosis was presumed to predate death by 6 months. If the duration was not given on the death certificate, but a diagnosis was recorded in another source, the earliest such date was used to determine the time to dementia. If sources had recorded cognitive decline, mild cognitive impairment and dementia, the date was taken as the earliest recording of a dementia diagnosis. If dementia was ascertained based on evidence within the records that did not include a formal diagnosis of dementia, the earliest mention of cognitive impairment was used to date dementia (as long as this did not specifically note the absence of a dementia syndrome). For those who did not develop dementia, the time to dementia variable was recorded as either the time to date of death or to a date beyond that last date of data collection for any surviving participant.

### Participant exclusions

Any participant reporting a history of dementia or scoring 23 or less on the Folstein Mini Mental State Examination [32] (MMSE) at baseline was excluded from our analyses. Those without a valid MMSE score at baseline were also excluded, as were those without any follow-up data available. To minimise the potential for classification error, possible dementia cases were excluded from the analyses and probable dementia was used as the primary outcome in this study.

### Statistical analysis

We first performed simple comparison analyses between the group who developed dementia and the group who did not, for each of the included variables. Univariate analyses were completed using either a Pearson chi-square (for categorical variables) or independent samples t-test (for continuous variables) (*IBM SPSS, Version 21)*. A *p* value of < 0.05 was used to demonstrate a statistically significant difference. All subsequent steps in the analyses were completed using the *R* statistical software (version 3.3.3) [33].

The second stage of the analyses used binary logistic regression to examine the potential risks for dementia associated with the fitness measures. In a first model (logistic regression model 1) we included the fitness variables along with those factors known to be associated with dementia in our cohort and the other important control variables (FEV_1_, grip strength, 6-m walk time, *APOE* ɛ4 carrier status, height, age, sex, history of hypertension, smoking status and age 11 IQ). The development of probable dementia was the outcome. In the second model (logistic regression model 2), the same variables were included, with the addition of a history of cardiovascular or cerebrovascular disease and a history of diabetes. A history of hypertension was considered separately to these other health variables as a statistically significant association with dementia had previously been observed within this cohort.

The main analyses used Cox regression models with death being included as a competing risk for dementia; in doing so, the influence of fitness on earlier death in the analysis of dementia development due to fitness was considered. The models were completed using standard software for Cox regression with right-censored data and treating death as censored, (R package survival). The first and second Cox regression models (Cox regression models 1 and 2) included the same covariates as those described for logistic regression models 1 and 2. Both models fitted the data acceptably with concordance of 64%. Analysis of the scaled Schoenfeld residuals (R function cox.zph), showed all covariates complied with the proportional hazards assumption. As is recommended practice, we supported the results of Cox regression with graphs demonstrating cumulative incidence for each competing event; illustrating the time-varying risk of dementia, between covariate levels. The unbiased estimate of cumulative incidence was calculated using the Aalen-Johansen estimator [34], (R packages prodlim and mstate).

## Results

### Participant eligibility

A total of *N* = 550 participants recruited to LBC1921 attended baseline testing at age 79 years. We excluded the following participants from the analyses: participants with an MMSE score of less than 24 at baseline (*n* = 9), participants without a valid MMSE score at baseline (*n* = 2), participants reporting a history of dementia at baseline (*n* = 2), and participants with no follow-up data available for the purpose of dementia ascertainment (*n* = 41). One additional participant was excluded as the calculated time to dementia suggested that dementia predated attendance at wave 1 testing. From the remaining participants (*n* = 495), a consensus diagnosis of probable dementia was agreed for *n* = 109. Those participants with a possible diagnosis of dementia were excluded from the analyses (*n* = 7).

### Participant demographics (Table 1)

The resulting eligible cohort (*n* = 488) included 280 females (57.4%) and 419 participants (85.9%) were known to be deceased [29]. This included 331 who died with no diagnosis of dementia. Descriptive statistics for those eligible for inclusion (both with and without dementia), and those excluded are shown in Table 1.

**Table 1:** Study Sample Demographics and Group Comparison

	Eligible Participants (*n* = 488)		Group Comparison *p* value (chi-square or t-test)	Excluded Participants (*n* = 62)
	Dementia (*n* = 109)	No Dementia (*n* = 379)		
Age	*n* = 109	*n* = 379		*n* = 62
-mean age in years (SD)	79.04 (0.55)	79.08 (0.59)	0.54	79.09 (0.53)
Sex	*n* = 109	*n* = 379		*n* = 62
-% female	62.4%	55.9%	0.23	58.1%
Living or deceased	*n* = 109	*n* = 379		*n* = 62
-% deceased	80.7%	83.3%	0.08	29.0%
MMSE score at baseline	*n* = 109	*n* = 379		*n* = 60
-mean score (SD)	28.10 (1.64)	28.33 (1.46)	0.16	27.27 (2.67)
Height	*n* = 106	*n* = 378		*n* = 60
-Mean height in cm (SD)	162.11 (9.21)	163.59 (9.45)	0.15	163.83 (8.53)
*APOE* ɛ4 carrier status	*n* = 109	*n* = 373		*n* = 61
-% carrier *APOE* ɛ4	41.3%	22.5%	*< 0.001*	27.4%
Age 11 IQ (standardised)	*n* = 101	*n* = 339		*n* = 53
-Mean score (SD)	100.19 (16.18)	100.22 (14.53)	0.98	98.21 (15.63)
FEV_1_	*n* = 106	*n* = 378		*n* = 60
-mean rate in litres per second (SD)	1.95 (0.59)	1.84 (0.62)	0.12	2.03 (0.68)
Grip strength	*n* = 106	*n* = 378		*n* = 60
-mean strength in kilograms (SD)	25.89 (10.17)	26.46 (8.87)	0.57	28.18 (8.59)
6 m walk time	*n* = 105	*n* = 377		*n* = 59
-mean time in seconds (SD)	4.56 (1.53)	4.84 (2.11)	0.20	4.37 (1.29)
Smoking status	*n* = 108	*n* = 379		*n* = 62
-% ever smoker	42.6%	61.7%	*< 0.001*	50.0%
History of cardiovascular or cerebrovascular disease	*n* = 104	*n* = 373		*n* = 58
-% positive history	28.9%	28.2%	0.89	24.2%
History of hypertension	*n* = 108	*n* = 375		*n* = 61
-% positive history	35.2%	41.9%	0.21	41.0%
History of diabetes	*n* = 109	*n* = 379		*n* = 62
-% positive history	4.6%	5.8%	0.62	1.6%

Italicized results demonstrate significance of p<0.05

### Event variables

The mean time to death for the *n* = 419 participants who were known to be deceased was 3144.5 days (SD: 1517.5). For those who survived, the time to death was taken as an arbitrary point beyond the last date of data collection for any participant; 6500 days. The mean time to dementia for the *n* = 109 who developed dementia was 3535.7 days (SD: 1283.3). A total of *n* = 379 participants remained dementia free; for these participants, time to dementia was taken either as the time to death for those who died (*n* = 331, mean = 2863.0 days, SD: 1469.4), or an arbitrary point beyond the last date of data collection for any participant (*n* = 48, 6500 days).

### Dementia group comparison (Table 1)

Also included in Table 1 are the results of the group comparison analyses. Univariate analyses demonstrated little difference between those participants who developed dementia and those who did not. Only smoking status (*p* < 0.001) and *APOE* ɛ4 (*p* < 0.001) carrier status demonstrated statistically significant difference (*p* < 0.05).

Univariate analyses were also performed to compare those who died with those who survived. Lower FEV_1_ and greater 6-m walk time were both associated with an increased risk of death (*p* = 0.02 and *p* = 0.01, respectively). The results for these univariate analyses are available in Additional file 1: Table S1.

### Logistic regression results (Table 2)

The results of both logistic regression analyses demonstrated that *APOE* ɛ4 remains an important risk factor for dementia after age 79 years (logistic regression model 2: Odds Ratio (OR) 2.52 (95% confidence interval: 1.50, 4.22), *p* < 0.001). Both initial models also suggested that increased FEV_1_ at age 79 increased the risk for subsequent dementia (logistic regression model 2: OR 1.93 (1.07, 3.57), *p* = 0.03). The only other variable that reached statistical significance was height, and only in the second model; increased height was shown to decrease risk for subsequent dementia (OR 0.95 (0.91, 1.00), *p* = 0.04).

**Table 2:** Regression Results

	Odds/ Hazard Ratios (95% CI) for Probable Dementia			
	Logistic Regression Model 1	Cox Regression Model 1	Logistic Regression Model 2	Cox Regression Model 2
	Odds Ratios (95% CI)	Hazard Ratios (95% CI)	Odds Ratios (95% CI)	Hazard Ratios (95% CI)
	(*n* = 425)	(*n* = 425)	(*n* = 416)	(*n* = 416)
Sex (female)	0.77 (0.31, 1.85)	1.26 (0.56, 2.83)	0.86 (0.34, 2.11)	1.42 (0.63, 3.23)
Age (days)	1.00 (1.00, 1.00)	1.00 (1.00, 1.00)	1.00 (1.00, 1.00)	1.00 (1.00, 1.00)
Height (centimetres)	0.96 (0.92, 1.00)	0.97 (0.94, 1.01)	*0.95 (0.91, 1.00)*	0.97 (0.93, 1.01)
*APOE* ɛ4 carrier	*2.47 (1.49, 4.08)*	*2.72 (1.78, 4.14)*	*2.52 (1.50, 4.22)*	*2.85 (1.85, 4.41)*
Age 11 IQ	1.00 (0.98, 1.02)	0.99 (0.98, 1.01)	1.00 (0.98, 1.02)	1.00 (0.98, 1.01)
History of hypertension	0.64 (0.38, 1.05)	0.79 (0.51, 1.23)	0.64 (0.38, 1.06)	0.78 (0.50, 1.22)
Smoker (ever)	0.63 (0.38, 1.04)	0.92 (0.60, 1.40)	0.62 (0.37, 1.03)	0.94 (0.61, 1.45)
FEV_1_ (l/s)	*2.05 (1.15, 3.75)*	1.43 (0.82, 2.48)	*1.93 (1.07, 3.57)*	1.30 (0.74, 2.30)
6 m walk time (s)	0.93 (0.79, 1.07)	0.99 (0.87, 1.13)	0.94 (0.79, 1.08)	0.99 (0.87, 1.13)
Grip strength (kg)	1.00 (0.96, 1.05)	0.98 (0.94, 1.02)	1.01 (0.96, 1.05)	0.98 (0.94, 1.02)
History of cardiovascular or cerebrovascular disease	–	–	1.08 (0.61, 1.86)	1.14 (0.72, 1.81)
History of diabetes	–	–	0.86 (0.19, 2.88)	1.39 (0.41, 4.66)

Logistic regression model 1 and Cox regression model 1: included FEV_1_, grip strength, 6-m walk time, APOE ɛ4 carrier status, height, age, sex, history of hypertension, smoking status and age 11 IQ, with the development of probable dementia as the outcome. Logistic regression model 2 and Cox regression model 2: as logistic regression model 1 plus history of cardiovascular or cerebrovascular disease and history of diabetes. Results for Cox regressions show the hazard ratios and 95% confidence intervals. Statistically significant results are italicized

### Cox regression analyses (Table 2)

In both Cox regression models, *APOE* ɛ4 continued to be an important predictor for dementia (Cox model 2: Hazard Ratio (HR) 2.85 (95% confidence interval: 1.85, 4.41), *p* < 0.001). The results did however demonstrate that once death was considered within the analyses, the association between FEV_1_ and dementia did not reach statistical significance in either model (Cox model 2: HR 1.30 (0.74, 2.30), *p* = 0.37). Neither grip strength (Cox model 2: HR 0.98 (0.94, 1.02), *p* = 0.35) nor walking speed (Cox model 2: HR 0.99 (0.87, 1.13), *p* = 0.90) was associated with dementia in either model. No other variable was demonstrated to be associated with dementia.

### Cumulative incidence graphs

**Fig. 1:**
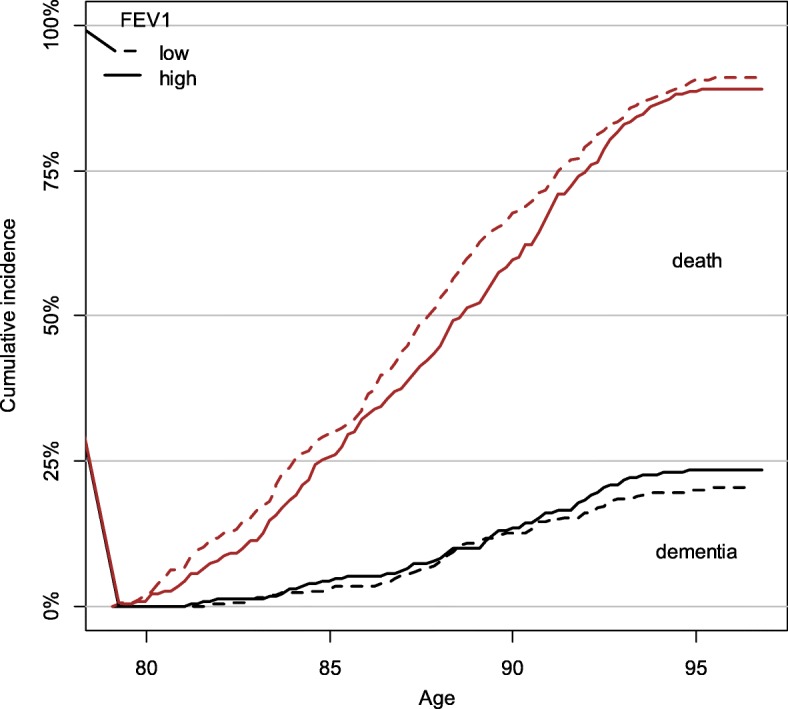
Cumulative incidence of dementia and death (stacked) at 2 levels of FEV_1_. Note. FEV_1_ groups were split at 1.8 l per second

**Fig. 2:**
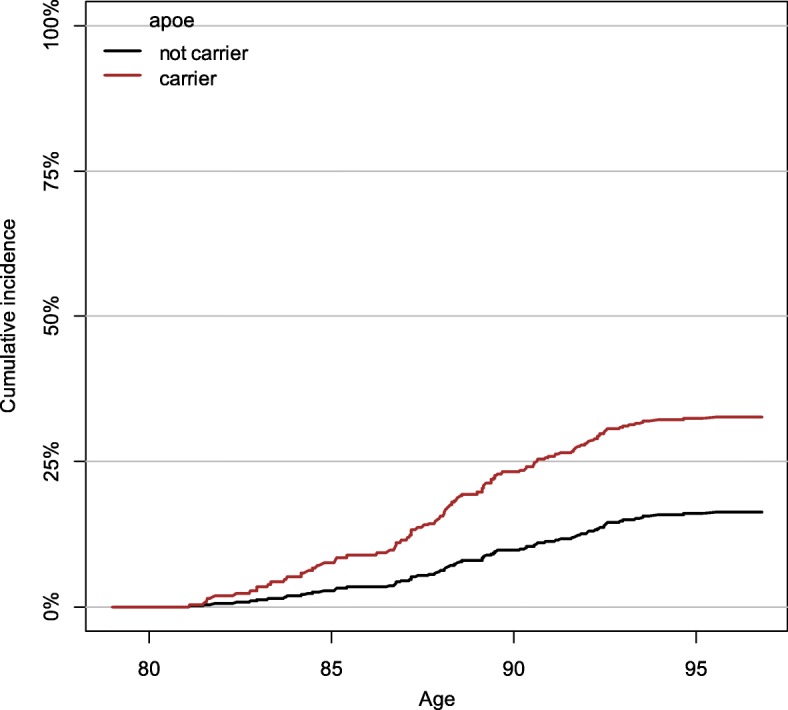
Cumulative incidence of dementia for *APOE* ɛ4 carriers and non-carriers. Note. Predicted cumulative incidence of dementia (Aalen-Johansen estimator) for men aged 79 of average height, fitness (FEV, 6 m walking time, and grip strength), and age 11 IQ

For the purposes of calculating cumulative incidence, FEV_1_ results were divided into two groups, at a value of 1.8 l per second. This value was close to both the sample mean and median. The stacked cumulative incidence plot shown in Fig. 1 demonstrated that increased FEV_1_ was associated with decreased risk of death but not dementia (Fig. 1). A second cumulative incidence plot confirmed that the presence of an *APOE* ɛ4 allele was associated with an increased risk for dementia (Fig. 2).

## Discussion

Contrary to the existing evidence within the literature [2–15], this study found that decreased fitness beyond age 79 was not a risk factor for subsequent dementia. More specifically, FEV_1_, grip strength and walking speed at age 79 years were not found to be associated with dementia. Indeed, considering those who survive, greater FEV_1_ is associated with an increased risk of dementia: that is, if you survive, having better respiratory function (a measure of fitness) when you are younger makes it more likely that you will develop dementia. Even when the effect of poor respiratory function on mortality risk is taken into account, being fitter as a younger adult does not confer any benefit with regards to dementia risk once you reach your ninth decade. This raises an important distinction because better physical fitness was associated with higher cognitive scores in oldest age, even after adjusting for childhood IQ, in this same cohort [1]. That is, having better physical fitness at age 79 was good for a person’s cognitive ability but did not reduce the risk of developing dementia. As was observed in a previous study of the LBC1921, the presence of an *APOE* ɛ4 allele was however associated with an increased risk for dementia [29].

Previous studies have repeatedly shown that poorer lung function, grip strength and walking speed are associated with increased risk for dementia [2–15]. The primary reason for this inconsistency may be that most previous studies have investigated the association in a younger population, or a population with a broader age range than the LBC1921 [2, 7]; the results of this study may differ simply because the results are specific to the oldest-old. We might also consider the impact of death on our finding. In our cohort, increased lung function at age 79 was associated with a reduce risk of death. We can therefore hypothesize that our null findings may be due to the fact that those with poorer fitness, who would have been more likely to develop dementia, would have died from another cause before developing dementia. Taking lung function as an example, in addition to death directly related to lung disease, death before dementia may occur because of diabetes, atherosclerosis or coronary heart disease, all of which are known to be associated with poorer lung function [5]. Given that our competing risks analyses accounts for this possibility, we would not expect to have found an increased risk of dementia for those with low FEV_1_ if these people had survived.

In examining the potential influences on our findings it is worthwhile considering the fitness levels of the participants of the study, when compared with population norms. While predicted values for FEV_1_ are less well established for those aged over 70 years, formulae published by the British Thoracic Society can be used to calculate the expected results for our study population [28]. Females included in our study achieved, an average, 94.5% of the predicted FEV_1_ value, while males achieved 88.8% of the predicted value, based on mean age and height. Both of these were above the 80% cut-off used to demonstrate abnormality, and below the 100% threshold that would demonstrate better than expected lung function. The mean walking speed recorded for male and female study participants (137 cm per second and 118 cm per second, respectively) were similar to published normative values for persons aged in their 70s (133 cm per second and 127 cm per second for males and females respectively) [35]. Previously published expected median values for grip strength – measured using a Jamar Hydraulic Hand Dynamometer – also closely resemble those achieved in the present study [36]. For males, the expected median was 34.9 kg, and the achieved median and mean were 34 kg and 34.5 kg respectively. For females, the expected median was 20.9 kg, and the achieved median and mean were 20 kg and 20.3 kg respectively. One might expect the values for grip strength and walking speed to be slightly below expected levels because the reference values were for individuals aged 70–79 years and the participants of this study were at the uppermost limit of this age bracket. Based on these results one might assume that, with respect to grip strength, walking speed and FEV_1_, this study cohort were not of unusually high or low fitness and reflected a typical population of this age.

It is also possible that the lack of association observed in our results is the result of some type of resilience to disease. It is acknowledged within the literature that some individuals do not appear to be susceptible to the negative effects of certain lifestyle habits. For example, some heavy smokers do not develop lung disease [37]. Studies – including a large Medical Research Council funded study of UK Biobank data – have suggested that DNA variants may explain why some individuals can have relatively good lung health despite smoking [38, 39]. It is possible that there is a similar unidentified genetic resilience for dementia, such that those who are more susceptible to developing dementia because of certain risk factors will do so prior to oldest age, while those who are genetically resilient will continue to be resilient in oldest age, resulting in a lack of observed association.

The null findings of this study should not be overlooked as unimportant. If these associations are indeed only present in earlier old age, the fact that lung function, grip strength and walking speed were not significantly associated with dementia in our cohort, demonstrates an important difference between the risk profile for dementia in early old age and late old age.

Since the median age of dementia diagnosis in the UK, for example, is now over 80, if our findings are replicated, it means that for the majority of people who will develop dementia seeking to improve physical fitness is unlikely to have any effect on preventing the disease. Although our findings do not concur with previous reports, our findings are supported by other health-related analyses in this cohort that identified changes in the risk factor profile for dementia in advanced old age [29]. In the LBC1921, a history of hypertension was associated with a decreased risk of dementia, and increased physical activity in early adulthood was associated with an increased risk for dementia after age 79 years [29]. Whereas the relationship between physical activity and dementia was novel, other studies have described a change in the relationship between hypertension and dementia in the oldest old [40]. The results of the present study, together with these previous findings, would indicate that there is different risk profile for dementia in the oldest old, when compared with those in earlier old age.

Our findings therefore highlight two important differences. Firstly, it seems increasingly likely that the risk profile for dementia in oldest age differs from the risk profile in earlier old age. Secondly, that the risk profile for dementia in oldest age differs from the risk profile for less successful non-pathological cognitive ageing in the oldest old. Understanding the risk profiles for each of these separate processes and how they differ is important, as this knowledge will aid in the design and development of appropriate prevention and management strategies. Given that dementia is set to be one of the greatest public health challenges facing the ageing population, successful cost-effective prevention strategies are of vital importance.

### Strengths and limitations

A significant strength of this study lies in the suitability of the cohort for these analyses. The minimal cultural, ethnic and geographical variability within the narrow-age cohort minimises confounding errors and means that the results are specific to the oldest old. We recognise, though, that this limits the generalisability of the results, and so additional studies are required in other groups. The availability of a childhood IQ score is a rare strength of the study, as is the detailed follow-up completed since enrolment in the study. The high mean baseline MMSE score (28.3, SD: 1.5) for those included in the analyses increases the likelihood that we identified incident cases of dementia, rather than prevalent cases. By including multiple measures of fitness it was possible to consider several components of fitness. Whilst this was a strength of our study, it could be argued that additional tests could have provided a more comprehensive assessment of fitness. We selected the additional variables for inclusion in the analyses based on the available evidence, but it is possible that there was some residual confounding due to a variable not considered in this study. It is not however possible to exclude all such possibilities without negatively affecting the analyses through multiple hypotheses testing. The dementia ascertainment method in this study has previously been shown to be effective and comparable with expected rates [29]. Having determined dementia cases retrospectively using existing data, it was not possible to accurately date the onset of dementia. For this reason, we cannot be entirely confident in the accuracy of the time to dementia variable. The method used to determine date of onset in this study, is however conservative and it is likely that dementia onset preceded our assigned date by some time. As such, the true competing risk of death is almost certainly less than we are adjusting for in the model. While we recognise this limitation of our study, it is not unique to our method; it is notoriously difficult to pinpoint the exact date of dementia onset, even in studies using prospective clinical follow-up. This is particularly true given that the most common form of dementia – Alzheimer’s disease – has a gradual onset. A further limitation of our study is the possibility of missed cases. Our results could also have been affected by using an outcome of probable dementia of any subtype. The number of participants with each subtype of dementia was too few to perform individual analyses. It is also possible that the overall size of our study cohort had an impact on our findings, and that a larger sample size would have produced differing results.

## Conclusions

The results of this study suggest that increased physical fitness at age 79 years does not reduce the risk for subsequent dementia. The early death of participants with poorer fitness, who would have been more at risk of dementia dying before developing dementia, might explain why the findings differ from studies of earlier old age.

## Additional file

Additional file 1: **Table S1.** Group Comparison: Deceased and Living. (DOCX 14 kb)

## Abbreviations

*APOE* ɛ4: Apolipoprotein E ɛ4
CHI: Community Health Index
CI: Confidence Interval
DNA: Deoxyribonucleic Acid
FEV_1_: Forced Expiratory Volume in 1 s
IQ: Intelligence Quotient
LBC1921: Lothian Birth Cohort 1921
MHT: Moray House Test
MMSE: Mini Mental State Examination
NHS: National Health Service
OR: Odds Ratio
PIMS: Patient Information Management System
SD: Standard Deviation
SMS1932: Scottish Mental Survey 1932
Trak: TrakCare
