# Supplementary material for: Physical fitness and dementia risk in the very old: a study of the Lothian Birth Cohort 1921
Source: BMC Psychiatry. 2018 Sep 4;18:285. doi: 10.1186/s12888-018-1851-3 (PMC6123983; doi:10.1186/s12888-018-1851-3)
Supplement: Supplementary file 1 — Table S1. Group Comparison: Deceased and Living. (DOCX 14 kb) [file 12888_2018_1851_MOESM1_ESM.docx]

Table S1. Group Comparison: Deceased and Living

|  | **Eligible Participants**  (n=488) | | **Group Comparison p value**  **(chi-square or t-test)** |
| --- | --- | --- | --- |
|  | **Deceased**  (n=419) | **Not Deceased**  (n=69) |  |
| **Age**  -mean age in years (SD) | n=419  79.07 (0.59) | n=379  79.12 (0.58) | 0.51 |
| **Dementia or no dementia**  -% deceased | n=419  21.0% | n=69  30.4% | 0.08 |
| **Height**  -Mean height in cm (SD) | n=415  163.29 (9.30) | n=69  163.13 (10.08) | 0.90 |
| **APOE ɛ4 carrier status**  -% carrier APOE ɛ4 | n=413  28.3% | n=69  17.4% | 0.06 |
| **Age 11 IQ (standardised)**  -Mean score (SD) | n=381  99.77 (15.18) | n=59  103.12 (12.74) | 0.11 |
| **FEV_1_**  -mean rate in litres per second (SD) | n=415  1.84 (0.62) | n=69  2.03 (0.59) | **0.02** |
| **Grip strength**  -mean strength in kilograms (SD) | n=415  26.26 (9.10) | n=69  26.75 (9.59) | 0.68 |
| **6 metre walk time**  -mean time in seconds (SD) | n=413  4.88 (2.10) | n=69  4.21 (1.10) | **0.01** |
| **Smoking status**  -% ever smoker | n=418  58.9% | n=69  49.3% | 0.14 |
| **History of cardiovascular or cerebrovascular disease**  -% positive history | n=411  28.7% | n=66  25.8% | 0.62 |
| **History of hypertension**  -% positive history | n=415  41.5% | n=68  33.8% | 0.24 |
| **History of diabetes**  -% positive history | n=419  6.0% | n=69  2.9% | 0.30 |

*Note: For results highlighted in bold, p<0.05*
